# The KT Jeang Retrovirology prize 2022: Florence Margottin-Goguet

**DOI:** 10.1186/s12977-022-00606-3

**Published:** 2022-09-06

**Authors:** Retrovirology Editorial

**Affiliations:** London, UK


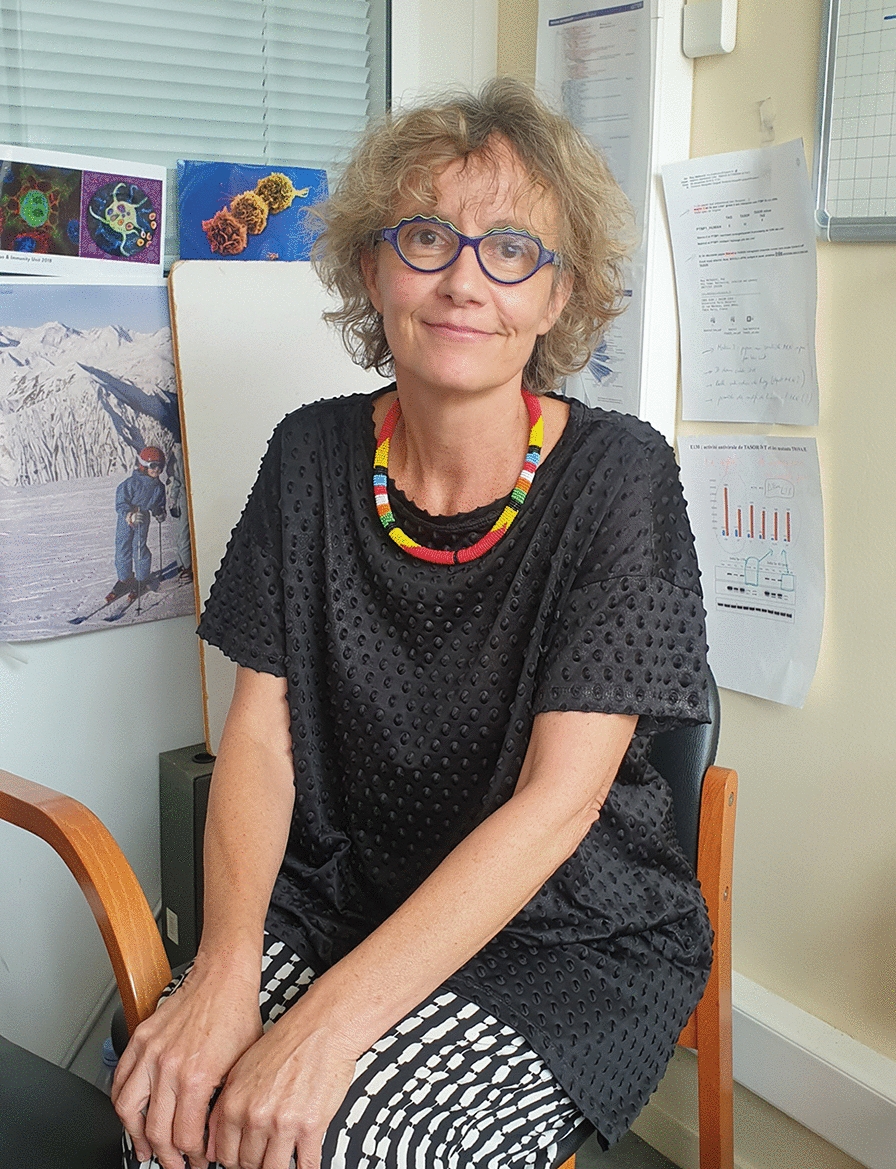
Florence Margottin-Goguet studied cellular and molecular biology at the University Pierre and Marie Curie in Paris. As a PhD student, she worked from 1989 to 1993 in the laboratory directed by André Sentenac in Saclay, near Paris. Sentenac's lab was internationally recognized in the field of transcription research using the *Saccharomyces cerevisiae* as a model organism. Specifically, the laboratory focused on the isolation of the essential components of the transcription machinery as well as on the elucidation of the mechanisms underlying transcription activation. Florence first worked under the guidance of Dr. Geneviève Dujardin and Dr. André Sentenac. She was involved in the discovery that TAT-binding factor (TBP) is required for the initiation of RNA polymerase 3-mediated transcription, and is therefore not only a cofactor of RNA polymerase 2 as was thought at the time [1]. Later on, TBP was found to be an essential factor in gene transcription regardless of the type of RNA polymerase involved.

During her doctoral studies, Florence also collaborated with Anne-Françoise Burnol. Together, they showed that TFIIIC, a multi-subunit complex required for the recruitment of RNA polymerase 3 to genes, was involved in the relief of transcription repression by chromatin [2]. This work, which was performed under the expert guidance of Geneviève Almouzni and Marcel Méchali, was Florence's first introduction to the regulation of gene expression in a chromatin environment. Altogether, at the beginning of her career, Florence experienced the common fate of biochemists: long hours in the cold room loading protein extracts onto various types of chromatography columns, harvesting numerous protein fractions which were subsequently monitored for their activity in in vitro transcriptional assays. During this period, she had the opportunity to meet fundamental research lovers such as Sylvie Camier, Geneviève Dujardin and Anne-Françoise Burnol. These researchers have definitely influenced Florence’s vision of research practice: hypothesis-driven studies and well-designed experiments are rewarded by lots of fun. During this time, Florence also met her future husband and, shortly after obtaining her PhD, they both joined the laboratory of molecular parasitology of the Institut Pasteur in Cayenne, French Guiana. In this lab, candidate vaccines against malaria were monitored. This period was a kind of parenthesis during which Florence tackled a totally different type of research. This stay in French Guiana was rich in discoveries both inside and outside the lab where she and her husband very much enjoyed moving up rivers by pirogue and discovering the beautiful rainforest. This was the first of many travels around the world, a second passion of Florence’s, besides research.

Back to Paris in 1994, Florence wished to return to fundamental research. She has always been convinced that basic research is a fertile ground for applied science, especially in the medical field. Accordingly, she chose to study HIV-1 and HIV-2 viruses, the etiological agents of AIDS, as well as related simian viruses (SIV). She obtained a post-doc fellowship from SIDACTION (a French association funding a wide range of research projects on AIDS) and joined the lab headed by Richard Benarous, at the Institut Cochin. This institute provided a stimulating environment as several teams were studying various aspects of the HIV virus biology. Richard Benarous was sharing his enthusiasm about a program he had developed to identify the host cell partners of a set of HIV proteins in order to elucidate their mechanisms of action. The so-called “two-hybrid screening” strategy was particularly well-suited to this approach and, in France, Richard's laboratory had been a forerunner in the development of this method, first described by Fields and Song [3]. Florence was assigned the search for partners of the HIV-1 Vpu protein, as a research project. She remembers her first steps in the cell culture room, as she had never cultivated cells before! She also remembers the harsh discussions between teams about the relevance, or rather lack of relevance, of two-hybrid screens in the field of virology!

It had long been shown that HIV-1 Vpu promotes the degradation of CD4, the cell receptor of HIV although the underlying mechanism remained elusive [4]. The two-hydrid screen performed by Florence yielded several Vpu-interacting candidates, including the human homolog of the Xenopus βTrCP protein. Thumbing through different papers in the nearby laboratory library, Florence discovered that human βTrCP shared a sequence motif with ubiquitin ligases subunits involved in the targeting of proteins to the ubiquitin-mediated proteolysis pathway [5]. Furthermore, in 1993, pioneering work of Martin Scheffner uncovered that the papillomavirus E6 protein uses an ubiquitin ligase to induce the degradation of the p53 tumor suppressor protein [6]. Taken together, these findings led Florence to hypothesize that Vpu bridges CD4 to βTrCP promoting its degradation by an ubiquitin-mediated pathway. Following numerous biochemical experiments demonstrating the existence of the Vpu/CD4/βTrCP ternary complex, the hypothesis was fully confirmed thanks to the collaboration with Klaus Strebel’s team at the NIH. The “Vpu-βTrCP story” has indeed provided the first example of ubiquitin ligase hijacking by an HIV auxiliary protein [7]. It has then turned out that hijacking of ubiquitin ligases is a method employed by almost all HIV auxiliary proteins and it is widely used by viruses to elegantly get rid of host factors detrimental to their life cycle. In this mechanism, a viral protein bridges a host ubiquitin ligase to a specific host protein which is not its natural substrate. The hijacking of ubiquitin ligase by HIV auxiliary proteins then became the favorite theme of Florence’s research.

βTrCP was the first identified human member of the family of F-box proteins which are adaptors of the Cullin 1 ubiquitin ligase complex. Each member of the family has its own specific targets. Still with the precious mentorship of Richard Benarous, Florence contributed to the identification of the first natural substrates selected by βTrCP for subsequent degradation: IκBα, βCatenin, ATF4, involved in cancer, inflammation and stress response, respectively [8–10]. A hint was the presence in these cellular substrates of a sequence similar to the βTrCP-binding motif present in HIV-1 Vpu, the “DSGXXS motif” (and derivatives). The presence of this linear motif became a very good indicator of βTrCP binding, leading to the identification of hundreds of βTrCP host targets. Based on this example, Florence likes to pinpoint how discoveries in virology may benefit other fields related to different diseases.

In 1998, Florence got a permanent position at INSERM (National Institute for Medical Research in France). In 2000, she moved to Palo Alto along with her husband and their young daughter to join the laboratory of Peter Jackson at Stanford University to tackle the cell cycle field. Going abroad for postdoctoral training after getting a permanent position was not common, but it was so wonderful: no pressure to get a position! But no pressure does not mean nothing happened. In two and a half years, Florence discovered a new mechanism for entering mitosis under the great mentorship of Peter Jackson. She also discovered the west coast of US through many trips in the beautiful national parks and gave birth to her second child at Stanford hospital. Her research project was to elucidate the mechanism of degradation of the mitotic inhibitor Emi1, a key step for entry into mitosis. When Florence left France, she did not anticipate that she would identify a βTrCP-binding motif in the Emi1 protein sequence but this finding definitively helped! βTrCP was soon identified as the ubiquitin ligase receptor of Emi1 [11, 12].

In 2005, Florence got her own lab, thanks to two grants dedicated to junior researchers, one called “Avenir” (literally the French word for “Future”) from INSERM and the other, from the city hall of Paris. She wished to take advantage of the knowledge gained from her previous work to study interference of HIV auxiliary proteins with cell cycle progression. Vpr, an auxiliary protein of HIV viruses, was known to cause cell cycle arrest specifically at the G2 phase since 1995 (reviewed in Ref. [13]). Florence decided to address two open issues regarding Vpr-mediated cell cycle arrest: the underlying mechanism and the significance for the viral cycle. Rapidly, she was joined by Catherine Transy and both co-directed the team until 2010. Florence and Catherine shared a common interest in the mechanism of ubiquitin ligase hijacking by viral proteins. As mentioned earlier, Florence had discovered that Vpu-mediated degradation of CD4 relies on its ability to recruit the Cullin 1 ubiquitin ligase through interaction with the βTrCP adaptor while Catherine showed that in vivo infection by hepatitis B virus requires direct interaction of HBx viral protein with DDB1, a core component of Cullin 4 ubiquitin ligase [14].

On the one hand, the team provided evidence that Vpu and Vpr viral auxiliary proteins could interact with two different ubiquitin ligases, being either adaptors or substrates of these enzymes [15, 16]. On the other hand, the team identified a missing link in Vpr-induced cell cycle arrest: the direct interaction between Vpr and DCAF1, an adaptor of the Cullin 4 ubiquitin ligase [17]. So far, only downstream steps in Vpr-mediated cell cycle arrest had been characterized.

Both Florence and Catherine were convinced that thorough scientific monitoring is often rewarded. They were first intrigued by Landau's group report that Vpr promoted the degradation of two uracil DNA-glycosidases involved in DNA repair and interacted with Cullin 1 and Cullin 4 ubiquitin ligase complexes [18]. Was it a clue that Vpr might hijack Cullin 1 or Cullin 4 ubiquitin ligases to promote the degradation of a host protein required for mitosis entry. Another hint arose from their routine habit to scrutinize any new publication regarding Cullin-based ubiquitin ligases. It turned out that among the recently described DCAF family of Cullin 4 ligase adaptors [19, 20] (others cited in Ref. [17]), DCAF1 had been previously isolated in 1994 by Zhao et al. as a Vpr-binding protein [21]! Everyone in the team rolled up their sleeves and evidence for DCAF1 being a critical player in Vpr-mediated cell cycle arrest quickly accumulated.

The results were published in “Cell Cycle”, after editors of other journals had rejected the manuscript before any reviewing process. At the time, Cell Cycle was a recent journal that advertised for quick review of “hot” manuscripts. It turned out that Florence’s paper was indeed very hot since five similar studies were published the same year [13]. However, among these published studies, only the Cell Cycle publication addressed whether interaction with DCAF1 was shared by Vpx, a Vpr-related protein found exclusively in HIV-2 and simian viruses. This being the case, it was intriguing: Vpx, in contrast to Vpr, showed no effect on cell cycle but was ascribed an essential role in productive infection of resting myeloid cells. Therefore, Florence and Catherine raised the hypothesis that during their evolution, Vpr and Vpx conserved the use of the DCAF1 ubiquitin ligase but diverged as for the selection of host proteins targeted for degradation, which in turn ensured distinct biological activities. The dependence of DCAF1 for Vpx activity was further demonstrated by the Skowronski and Stevenson’s groups, and then by Florence’s team in collaboration with Gianfranco Pancino’s team [22–24]. Florence loves telling this “Cell Cycle story”: journal with a small impact factor, but high impact in the field as shown by the strong competition that followed! Nonetheless, the team encountered some difficulties for recognition and funding; the steamroller of journal impact factors was harmful! Fortunately, the two AIDS French agencies, SIDACTION and ANRS, kept providing financial support.

The current view was (and still is) that during the so-called arms race between a virus and its host, the latter evolves endogenous factors that restrict productive infection sometimes very potently. Under this selective pressure, the virus in turn evolves countermeasures to escape host restriction. In the HIV field, Malim's group was a pioneer in the identification of such a host-virus relationship: the Vif auxiliary protein targets APOBEC3G for degradation thus escaping APOBEC3G-mediated editing of the viral genome [25, 26]. The host proteins that provide innate defense against viruses are termed “restriction factors” [27].

As already mentioned, the Vpr/Vpx field became highly competitive, several teams aiming at identifying Vpr and Vpx host targets which were expected to be new restriction factors.

In other words, the Vpr/Vpx treasure hunt had begun! Florence remembers discussions at the bar during the Cold Spring Harbor Laboratory meetings to get clues about what could be the myeloid restriction factor (degraded by Vpx) or the G2 arrest target (degraded by Vpr).

At the time, the “Tap-Tag” (Tandem affinity purification) was considered the method of choice to identify cellular interacting partners of a given (viral) protein. Using this method, her team succeeded in pulling down all subunits of the E3 ubiquitin ligase hijacked by Vpr but found no convincing host substrates.

In the meantime, in 2009, the “dNTP hypothesis” came out, thanks to Catherine: what if Vpx could enhance macrophage infection by increasing the pool of deoxynucleotides?

Again, surveying the literature, rather old in this case, was inspiring: it was shown that in resting myeloid cells -where Vpx operates- the short supply in deoxynucleotides (dNTP) was a limiting factor in HIV-1 infection [28, 29]. On the other hand, elegant work from the Cimarelli's group showed that addition of viral pseudo-particles containing SIV Vpx into the cell culture medium strongly enhanced dendritic cells infection by HIV-1 [30]. It was an unexpected and intriguing observation given the absence of a Vpx gene in the HIV-1 genome. Experiments conducted by Florence’s team soon confirmed that when exogenously provided, dNTPs and Vpx had very similar effects i.e. dramatic increase in the number of infected cells and in the speed of the reverse transcription step as well as the ability to act on distinct viruses.

The study was in progress to demonstrate that Vpx increased the endogenous pool of dNTPs in macrophages, thanks to the expertise of Beak Kim. However, the team was taken aback when Benkirane’s and Skowronski’s groups reported the identification of SAMHD1 as the myeloid restriction factor targeted for degradation by Vpx during the Cold Spring Harbor Symposium in 2011 [31, 32]. The team then immediately noticed that SAMHD1 displayed a HD domain suggesting a dNTPase activity which would support their initial hypothesis. The same year, two studies demonstrated the actual dNTPase activity of SAMHD1 in vitro [33, 34]. Rushing to perform in vivo experiments, the team of Florence, and those of Kim, Benkirane, Landau and Canard conjugated their efforts. Together they showed that SAMHD1 depletes the dNTP pool thereby inhibiting the synthesis of viral DNA in macrophages and that Vpx-mediated degradation of SAMHD1, increases the nucleotide pool, abrogating the restriction [35]. This seminal work received lots of attention in the HIV community, and beyond: SAMHD1 restricts other viruses/pathogens than HIV (some replicating in non-dividing cells via a DNA intermediate) and is a player in carcinogenesis through several mechanisms including its ability to modulate dNTP levels [36]. Later on, Florence put forward the hypothesis that SAMHD1 could be responsible for the early antiviral effect of interferon alpha. However, this was not the case as shown simultaneously by Florence’s team and Malim’s team in Retrovirology [37, 38].

In 2012, Florence and Claudine Pique merged their teams to create a new research unit at Institut Cochin, which was named “Retrovirus, Infection and Latency”. The team focuses on the two human pathogenic retroviruses, HIV and HTLV-1, the latter being the only human retrovirus associated to cancer development, specifically adult T-cell leukemia.

Florence's team pursued the idea of identifying new restriction factors. At the time, Florence thought that the role of Vpx was nearly fully understood. In striking contrast, the genuine function of Vpr in viral infection as well as the precise mechanism of Vpr-mediated cell cycle arrest remained to be elucidated: the relevant host proteins targeted for degradation by Vpr were still missing. Therefore, Florence did not want to give up on Vpr and kept in mind that a given viral protein may be endowed with the ability of targeting several host proteins thereby gaining distinct functions beneficial for the virus.

However, none of the potential Vpr host targets isolated from large scale immunoprecipitations and two-hybrid screens met the expected criteria [such as its depletion triggers G2 arrest and provides an advantage to the virus in macrophages]. Therefore, the team turned to another method termed SILAC (stable isotope labelling by amino acids in cell culture). This method aims at identifying proteins whose levels significantly vary in response to specific treatments, here incubation with viral pseudo-particles containing Vpr. Treatment with Vpx-containing viral pseudo-particles was meant as a control addressing both sensitivity and specificity of the method. Results were encouraging since SAMHD1 was not only retrieved but showed the best decrease ratio [39].

However, SILAC produced very few candidates for Vpr-induced degradation in contrast to the observations recently published by Lehner’s group [40]. Nonetheless, Florence's team identified the HLTF DNA translocase as a direct target of Vpr-induced degradation, which was simultaneously reported by Skowronski’s group [41, 42]. Somewhat disappointedly, HLTF downregulation failed to cause G2 arrest as expected for a direct player in this Vpr-activity. Thereafter, several cellular proteins were described as degraded by Vpr, some involved in Vpr-mediated G2 arrest, though the overall picture has remained unclear [40, 43, 44].

While Vpr gave everyone strong headaches, the surprise came again from Vpx. A master student in Florence’s lab, Ghina Chougui, exempted from benchwork because of a painful hand tendinitis, was given the project to reclassify the numerous SILAC data generated by the lab according specific criteria. The results highlighted that a host protein, FAM208A (later renamed TASOR), ranked just behind SAMHD1. This protein was previously retrieved in a screen designed to identify new HIV-1 restriction factors by the McKnight’s group [45]. Right away, Florence hypothesized that TASOR was a restriction factor counteracted by Vpx: TASOR combined antiviral activity with the property of being targeted for degradation by a protein known to use this mechanism against SAMHD1 restriction. The team quite easily confirmed Vpx-mediated degradation of TASOR but lacked clues as for the advantage it might confer to the virus. A corner of the veil was lifted in 2015 when the HUSH complex composed of TASOR, MPP8 and periphilin was reported by the Lehner's group to mediate epigenetic silencing of the HIV-1 provirus in a model of latency as well as silencing of cellular genes and retrotransposons [46]. Using this HIV-1 latency model, Florence’s team showed that Vpx as well as the genetically related SIV proteins was able to reactivate the latent virus through HUSH degradation [39]. Thanks to the expertise of Lucie Etienne, phylogenetic and evolutionary analyses highlighted that HUSH antagonism is lentiviral species-specific, which is typical of molecular arms race between restriction factors and viral antagonists, and identified signatures of potential “genetic conflicts” such as multiple isoforms in HUSH [39]. Altogether, on the host side, HUSH has emerged as a key player in the innate response against pathogens, whereas on the virus side, it has come as a surprise that the role of Vpx in viral escape from host restriction is not confined to resting cells. The team has again experienced harsh competition in the area since nearly at the same time, two other groups published similar conclusions about HUSH being a new target of lentiviral proteins [40, 47].

Today, the team is working on the mechanism of HUSH-mediated restriction. Roy Matkovic, a post-doc in the lab, is leading this project. The team has recently discovered that HUSH connects the deposition of repressive epigenetic marks on a HIV-derived transgene to the recognition and degradation of the nascent transcript, describing for the first time in mammalian cells a repressor complex involved in both RNA degradation and epigenetic silencing [48]. Another research axis is currently explored: is HUSH involved in virus persistence in the so-called reservoir cells in HIV carriers [49]? This project stands on a collaboration with a group of several physicians and medical virologists led by Véronique Avettand-Fenoel that recently joined Florence's team. Last but not least, Florence cannot help wondering whether the treasure hunt for Vpr and Vpx targets is over or is just at its beginning… Let’s see!
